# Neural stimulation systems for the control of refractory epilepsy: a review

**DOI:** 10.1186/s12984-019-0605-x

**Published:** 2019-10-29

**Authors:** Matthew D. Bigelow, Abbas Z. Kouzani

**Affiliations:** School of Engineering, Deakin University, Geelong, Victoria 3216 Australia

**Keywords:** Refractory epilepsy, Closed loop stimulation, Biomarkers

## Abstract

Epilepsy affects nearly 1% of the world’s population. A third of epilepsy patients suffer from a kind of epilepsy that cannot be controlled by current medications. For those where surgery is not an option, neurostimulation may be the only alternative to bring relief, improve quality of life, and avoid secondary injury to these patients. Until recently, open loop neurostimulation was the only alternative for these patients. However, for those whose epilepsy is applicable, the medical approval of the responsive neural stimulation and the closed loop vagal nerve stimulation systems have been a step forward in the battle against uncontrolled epilepsy. Nonetheless, improvements can be made to the existing systems and alternative systems can be developed to further improve the quality of life of sufferers of the debilitating condition. In this paper, we first present a brief overview of epilepsy as a disease. Next, we look at the current state of biomarker research in respect to sensing and predicting epileptic seizures. Then, we present the current state of open loop neural stimulation systems. We follow this by investigating the currently approved, and some of the recent experimental, closed loop systems documented in the literature. Finally, we provide discussions on the current state of neural stimulation systems for controlling epilepsy, and directions for future studies.

## Introduction

Epilepsy is a serious and debilitating disease, affecting more than 50 million people worldwide [1]. Of these, approximately one third suffer from refractory epilepsy, or epilepsy that cannot be controlled by medication. The uncontrolled nature of this disease means those affected sustain different levels of secondary injuries, illnesses, social dysfunction, and decreased life expectancy. Patients often experience a reduced quality of life (QOL) and the social stigma surrounding epilepsy often leads to discrimination and social rejection [2].

Resective surgery and newly developed or experimental drug treatments have been found to help around 65% of these patients [3]. For nearly 6.5 million sufferer’s, however, alternative treatments such as neural stimulation can be an effective, though potentially underutilised form of control. Neural stimulation has traditionally been in open loop form however, the use of closed loop systems, such as Cybernetics’s responsive neuro stimulation (RNS) or Neuropace’s vagal nerve stimulation (VNS), offers the potential for enhanced timing of delivery over open loop systems. However, despite this potential, when comparing to open loop systems, there has not been significant improvement in responder rates from those with a greater than 50% reduction in seizures using these methods. Leveraging research to provide an improved online prediction or detection method and integrating it into a system that can deliver effective, timely stimulation could be a step forward in in the battle to provide relief to these patients.

A successful closed loop system requires the interfacing of two subsystems: (i) a biosensing system that couples with signal classification algorithms to determine the epileptic state of a patient, and (ii) a neural device that provides effective control of seizures. Furthermore, these subsystems need to be interfaced using an appropriate communications protocol and a control algorithm to deliver an effective neural treatment using closed loop techniques. Several studies have shown that there are alternative options available in terms of biomarkers and classifiers that can be used to determine ictal and pre-ictal periods than those currently used in approved systems. Furthermore, there are alternative non-invasive neural stimulation systems other than the invasive methods currently approved for medical use. Therefore, in this paper, we have conducted a systematic literature search on Google Scholar, Pubmed and Medline, and IEEE Xplore using various combinations of keywords including *epilepsy, biomarkers, prediction, detection, closed loop, open loop, neural* and *stimulation*. Using the information derived from these searches we give a brief description of epilepsy, some of epidemiology surrounding it, and a look into what considered as refractory epilepsy. We investigate, and give examples of, some of the more important, biomarkers that can be used in the prediction and detection of seizures. These descriptions include the method of feature extraction and the algorithms used to classify pre-ictal and ictal periods. The paper then explores neurostimulation methods both currently in use and under investigation. These methods are categorised into invasive and non-invasive approaches showing that non-invasive approaches have the potential for further development in the future. Next, current approved closed loop systems are highlighted and described, followed by a description of some alternative methods that have been investigated to improve the closed loop systems. Finally, discussions are presented on the current state of the available systems, and suggestions are made for possible future directions that studies could take to improve the current systems. These suggestions could improve the overall quality of life of those suffering from this debilitating and potentially life-threatening condition.

## Epilepsy

### Description and epidemiology

Epilepsy is described [4] as a “disease of the brain characterized by an enduring predisposition to generate epileptic seizures, and by the neurobiologic, cognitive, psychologic, and social consequences of this condition”. Under normal circumstances, clusters of nerve cells called neurons interact electrically with other neurons to produce normal motor and non-motor actions by electrical and chemical signals. Seizures are caused by clusters of these neurons, signalling excessively and hyper synchronously [5]. Epileptic seizures are a consequence of these neurons producing these signals rapidly, and simultaneously manifesting in involuntary and spontaneous movements, emotions and behaviours, and sufferers can either be in a conscious or unconscious state [6–8]. This abnormal neuronal firing is what distinguishes epileptic seizures from nonepileptic events [5].

In 2015, epilepsy was responsible for more than 8 million years of life lost, and 14.6 million disability-adjusted life-years lost [9], both these figures are more than 4 times that of Parkinson’s disease. Despite technological advancement in traditional methods of treating epilepsy, including medication based treatments and surgical techniques, standardised mortality ratio and mortality rates have only decreased slightly in people diagnosed with epilepsy over the last 50 years [10].

### Refractory epilepsy

Refractory or drug resistant epilepsy accounts for about one third of all epilepsy cases [2]. ILAP’s definition of drug resistant epilepsy is: “drug resistant epilepsy may be defined as failure of adequate trials of two tolerated and appropriately chosen and used anti-epileptic drug (AED) schedules (whether as monotherapies or in combination) to achieve sustained seizure freedom” [11]. The uncontrolled nature of the disease can present those affected with increased experiences of additional illnesses. It is common for patients to experience psychological dysfunction which can result in reduced quality of life (QOL) and decreased life expectancy. Social stigmatisation commonly surrounds epilepsy and can lead to discrimination and social rejection [2].

The most common form of refractory epilepsy is temporal lobe epilepsy (TLE). TLE is a focal onset epilepsy that can become more generalized as the seizure progresses. There are two types of TLE, medial and neocortical. Medial, the most common, involves the internal structures of the temporal lobe, often beginning in the hippocampus or structures surrounding it. Neocortical is associated with the outer structures of the temporal lobe [12]. Treatment of this type of epilepsy ranges from continued medical treatment that includes the use of new or experimental drugs, which is estimated to eventually help approximately 15% of patients, and surgery including resective and non-resective procedures which leave an estimated 57% of patients seizure free [3]. The remaining 28% of drug-resistant epilepsy sufferers are without effective treatment and potentially benefit from alternative treatments such as neurostimulation.

## Epilepsy seizure prediction and detection

Identifying and capturing information of biometric markers of epilepsy is an area that could have a profound effect on expediating our knowledge and potentially engineering solutions for those with drug-resistant epilepsy. Clinically, little is still known about epilepsy biomarkers, for several reasons [13], however, research into identifying potential biomarkers for use in prognosis, diagnostic and seizure detection or prediction systems has been a focus of research for several decades [13, 14].

Most seizure tracking is currently conducted by patient recall and family experience, which is found to be unreliable due to the patient’s awareness during seizures [15]. The ability to autonomously predict or track seizures potentially have a large effect on an epileptic patient and their family’s quality of life. It has the potential to fill the gap for those drug-resistant epilepsy sufferers that have found no respite either in new AEDs or from surgical attempts to relieve their seizures. Neural stimulation procedures could benefit greatly from the ability to predict or track seizures potentially totally relieving those patients of symptoms.

Introducing warning systems, utilising the ability of biomarkers to identify seizures and wireless technology, could potentially lower mortality rates. Alerting patients, relatives or caregivers, that a seizure is imminent would allow them to take appropriate steps to limit the risk of further injury during high seizure risk periods. Moreover, continuous recording of biomarker activity can potentially help researchers with important clinical information that is not available to them by traditional reporting and tracking methods.

However, the balance between sensitivity and accuracy in seizure identification or prediction is an important consideration for any model. The trade-off between the two must be considered in the engineering of a closed loop neural stimulation system. Other considerations for biomarkers must also include the invasive/non-invasive requirements of the device. Invasive devices have risks including infection, rejection or procedural dangers, while consideration of non-invasive devices include the wearability, comfort and robustness of the device and the social stigma associated with wearing certain devices.

### Electroencephalogram (EEG) and electrocorticography (ECoG)

EEG or ECoG is perhaps the most researched form of seizure prediction and detection, and is part of the gold standard method used in clinical seizure detection. Electrical activity of the brain measured by EEG or ECoG, also known as intercranial EEG (iEEG), has produced potentially useful results in seizure prediction and detection. The combination of EEG/Video monitoring is the gold standard in seizure detection, used by clinicians internationally for epilepsy diagnosis [16]. EEGs is conducted by non-invasive methods consisting of placing several electrodes directly onto the scalp, usually 19 recording, one ground and a reference electrode in accordance with the international 10–20 system. On the other hand the ECoG is an invasive method that requires electrodes to be placed directly onto the brain’s surface [17]. An Australian ground-breaking, first-in-man, proof of concept study revealed sensitivities between 65 and 100% using intercranial EEG methods for seizure prediction in 15 patients, however, the algorithm used was unspecified in the literature [18] .

**Fig. 1:**
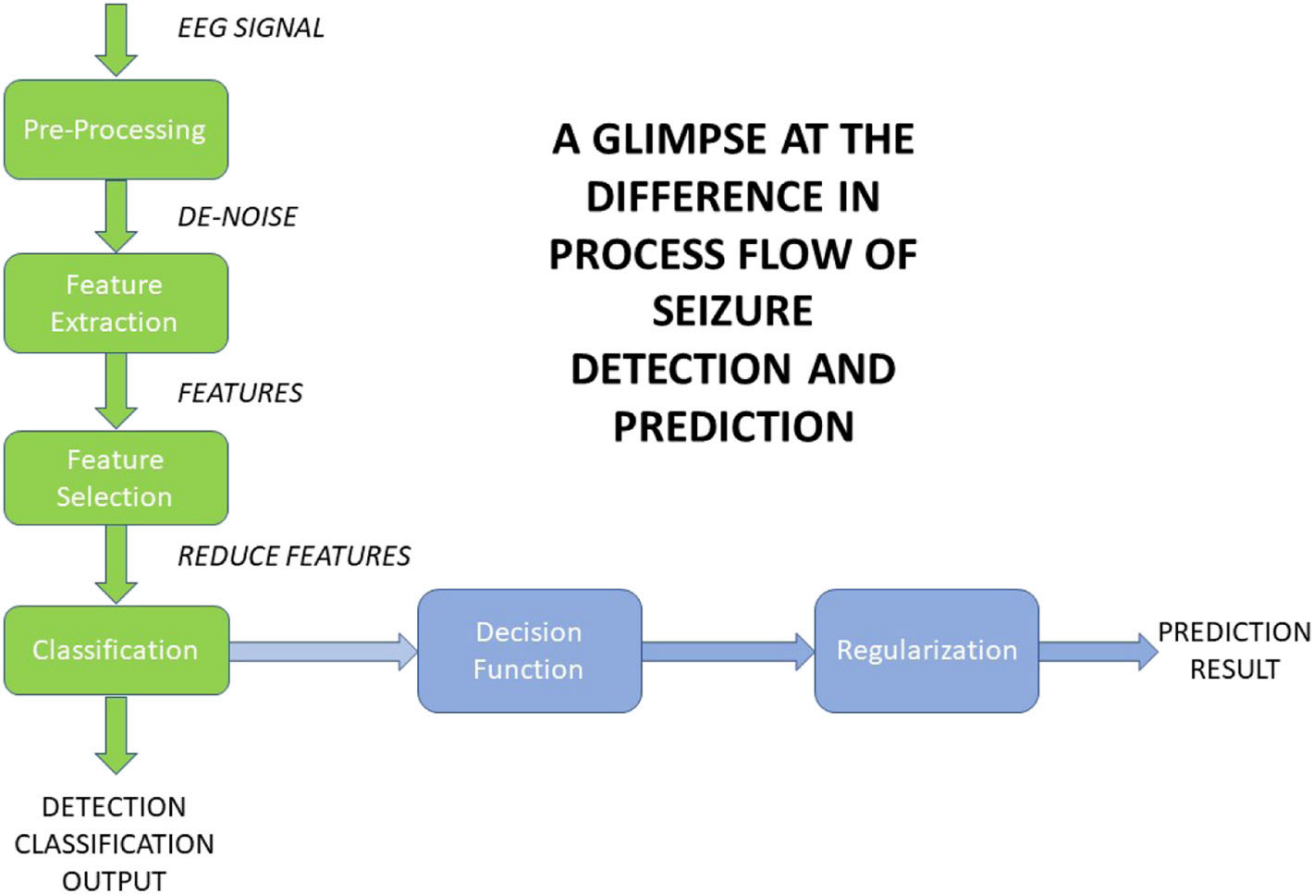
Basic Seizure detection flow [19]

Both methods result in a graphical record that is a measure of electrical activity in terms of voltage fluctuations in different areas of the brain. To allow seizure detection or prediction, these measurements are processed by taking several steps to extract features, analyse those features and then classify the signal appropriately. There are, however, significant differences in the steps required to complete seizure detection than those required for prediction [19] as shown in Fig. 1. A significant challenge with these methods is that seizure patterns can differ significantly from patient to patient, therefore any algorithm must be able to adapt to an individual’s pattern to successfully classify events [17, 20].

While methods used to detect seizures or predict ictal and preictal states have been classified as linear or non-linear methods, [17] suggests that due to a significant quantity of recent research focused on non-linear methods, an improved means to classify them is by their transform domain of operation.

### Seizure detection

In 2013, an 8-channel, ultra-low power, system on chip (SoC) seizure detection system was proposed by [20]. The system takes advantage of up to 120 s of stored EEG data, extracts features and then uses a machine learning seizure classification processor. The approach estimates the signal sub-bands, as opposed to the signal as a whole, to build a feature vector to detect seizure and non-seizure periods. The processor was “tested with the CHB-MIT database, and the SoC was verified with a rapid eye blink test, which shows typical accuracy of 84.4% with 2.03 μJ/classification energy efficiency” [20].

Tessy [21] used two time-domain features extracted from intracranial and scalp EEG databases. The method involved using both line length and energy feature extraction followed by a K nearest neighbour learning algorithm as the classification method for seizure detection. The method was described as simple, with lower costs and faster results when compared to more computationally expensive methods. It was found to give similar high degrees, nearing 100%, accuracy, specificity and sensitivity. It must be noted however that the datasets used for these methods were artefact free and would need to be tested under real world conditions [21].

Other methods include extracting statistics such as zero-crossing, entropy, mean or variance from time domain signals, and employing these in detection algorithms for the detection of seizures [17]. Alternatively, it has been suggested that wavelet features are the most commonly used features for seizure detection, however, more processing steps are required to extract features from wavelet coefficients, making it more computationally expensive than other methods [22]. An example of leveraging the wavelet domain was proposed in [23] when the authors produced a seizure detection system that uses only seven features obtained from, what is described as, a new class of minimally mean squared frequency localized, orthogonal wavelet filter bank designed for minimising the frequency spread. The method is shown to produce accuracies near 99% with sensitivities and specificity greater than 98%.

### Seizure prediction

Seizure prediction can be observed as a detection of the pre-ictal state that requires a considerable inter-ictal dataset to produce usable results [24]. proposed the use of a moving window analysis of positive zero-crossing intervals from scalp EEGs as the basis of the features. Specifically, using sets of reference distributions, the algorithm monitors changes over time of the distribution of positive zero-crossings. Using EEG data taken from 3 patients, it was found that the algorithm predicted 12 out of 14 seizures (86%) with an average time to seizure of 20.8 min and with a false positive rate (FPR) of 0.12 per hour [24].

In another approach, following pre-processing to remove certain artefacts, power features were extracted from 9 different spectral bands from 6 electrodes in each 20-s long half overlapping window. The method used a patient-specific classification algorithm based on a cost-sensitive support vector machine that could distinguish preictal from interictal states with a high sensitivity of near 80%. Also, importantly, the sensitivity was achieved with a zero FPR [25].

Behnam [26] proposed a real time prediction algorithm using recursive least squares (RLS) filtering. They proposed a real-time seizure prediction algorithm that included a Bayesian classifier and Hunting search to choose optimal features to train an offline seizure detection algorithm. A multi-layer perceptron classifier was trained with the pre-mentioned optimal features for an online detection algorithm, the RLS Filter was then applied to consecutive samples to gain online prediction. The algorithm was found to have an accuracy of 86.56%, precision rate of 86.53%, recall rate of 97.27%, FPR of 2.15 × 10^− 3^ per hour with a prediction time of 6.64 s.

Moreover, frequency methods were used in [27] in a field-programmable gate array (FGPA) based system for real time seizure prediction. A feature vector was extracted from six channels of iEEG data using a three-second sliding window with 2 seconds overlap, and computing the average cross spectral density of each pair of windowed signals, and the autospectrum density of each signal using Welch’s method. The brain was then modelled as a fully connected graph represented as a matrix using the resulting signals to weight the edges with the magnitude squared of a coherence estimate. The matrix was normalised and transformed to a ‘number of electrodes’ × 1 feature vector, representing the window by computing an element of its eigenvector centrality. The feature was then classified using a one-class support vector machine that classifies each feature as either normal or an outlier. This resulting output was used to further calculate the probability that a seizure is occurring by comparing a maximum likelihood estimate to a patient specific probability threshold. The algorithm successfully predicted 11 of 11 seizures with an average FPR of 3.9 per hour, at an average of 3.6 min before seizure onset [27].

In [28] investigated several seizure prediction and detection algorithms using wavelet components. The most successful method, when tested on adult seizure data, firstly used multiscale principal component analysis to denoise the EEG data, then wavelet packet decomposition was used to decompose the signal into wavelets. Four features were extracted from the resulting sub-bands including: (i) the mean of the coefficients’ absolute values in every sub-band, (ii) the average power of the coefficients in every sub-band, (iii) the standard deviation of the coefficients in every sub-band, and (iv) the ratio of absolute mean values of adjacent sub-bands. These features, using a random forest classifier, obtained an accuracy and sensitivity of over 99% for both seizure databases used in experiments. Although these results are high, the wavelet domain analysis can require high computational cost.

### Electrocardiogram methods

Epilepsy is believed to affect the cardiovascular function, and has been a focus of research over the last decade into the possibility of using heart rate variability (HRV) as a means of seizure prediction. The disproportionate neural activity that occurs in the preictal period has also been found to affect the area of the nervous system that affects the control of autonomous bodily functions including HRV [29].

Heart rate variability deals with the analysis of intervals between the beats of the heart. These intervals, known of RR intervals can be tracked and collected using an ECG to produce RR time series data [30]. This statistical information can then be leveraged to become the basis of analysis to predict oncoming seizures using methods like those discussed in EEG seizure prediction. This beat to beat analysis generally consists of several stages including: (i) pre-processing/filtration, (ii) feature extraction, (iii) feature selection stage, (iv) classification stage, and (v) validation stage. These stages consist of similar filtering and machine learning techniques as those discussed previously. Similarly, they are generally patient specific, requiring datasets to be established [31].

However, an advantage of ECG as a use for extracting a prediction or detection biomarker is that the non-invasive version is concealable for everyday use, thus it can potentially reduce the social stigma that would be associated with the wearing of an EEG monitor. In fact, [32] proposed the design of a wearable telemeter, compatible for RR Interval measurement that can operate for up to 10 h with the ability to store data in a smartphone via Bluetooth wireless transmission.

### Seizure prediction

Fujiwara [33] proposed a prediction method that uses multivariate statistical process control (MSPC), using features extracted from the HRV data. The method uses 8 features from the time and frequency domains and then uses MSPC to identify the preictal period as an anomaly from the usual interictal period. Tests were conducted on the clinical data from 14 patients that included 57 h of interictal data in which 8 patients had 11 awakening preictal episodes. Although it was noted that HRV individuality was important for prediction success, the proposed method demonstrated that 91% of preictal episodes could be predicted before seizure onset, with an FPR of approximately 0.7 times per hour where the preictal period was defined as a period within 15 min before a seizure occurs. It was suggested that although this is a higher FPR than that demonstrated by EEG methods, the method had greater advantage from an everyday use standpoint [33].

In [29] they proposed a prediction algorithm using the HRV analysis, and anomaly detection one-class support vector machines (SRV). The method uses extraction of the same 8 features from both the time and frequency domains of a window size, then analyses a matrix of eigenvalues and eigenvectors to produce 9 eigenvalues and principal components to be used in the classifier. The classifier separates the dataset into interictal and preictal parts, the interictal parts consist of the last 8 moving windows before 2 preictal intervals. Since there is a large amount of data for the interictal period, an anomaly detection approach is used and considers any interval that is not interictal, as preictal. The one-class SRV is used as the anomaly detection machine, it is an unsupervised machine learning algorithm that classifies new data as similar or different from the training set [29]. Tests of differing sized window periods were conducted on 31 sleeping patients with both generalised and focal onset seizures that had a total of 232 preictal intervals with a total of 85 h of interictal periods. Results showed that the optimal window size for prediction was 400 s with 100% sensitivity, a specificity rate of 92%, and precision rate of 92%. An F1 score of 92% was achieved with a fraction error length of 5% [29].

Another study analysed 8 HRV features to predict seizures from 7 patient’s data. The analysis results showed that linear and non-linear HRV features, such as mean heart rate, the relationship between SD1 (describes beat-to-beat rapid changes) and SD2 (describes long-term beat-to-beat changes), and the relationship between high frequency and low frequency components all changed during seizure episodes indicating that these parameters are useful in seizure detection. Moreover, a seizure prediction algorithm was developed where non-linear and linear features were extracted from uniform segment lengths and compared using a patient-specific threshold to previous segments. The algorithm resulted in 88.3% sensitivity and 86.2% specificity [34].

In a novel approach, [31] proposed a method for a non-patient specific solution tested on 18 recordings from 14 patients. The approach involved using RR time series data that had undergone specific pre-processing techniques and was windowed with 50% overlapping. For every window, 112 HRV features were extracted and an SVM with radial basis function kernel was used as a classifier. The data was classified into two classes, interictal or preictal. The results showed that a specificity value of 0.7252 and sensitivity of 0.7252 could be obtained for a window size of 7.8 min for which the features are calculated and 25.8 min for the preictal period.

### Seizure detection

As described previously, [34] describes features that can be used to distinguish seizure from non- seizure periods. Their method also specifies that the detection can be seen 5–10 min before the seizure onset, doubling as a short delay prediction method.

Similarly, [35] proposed a method of detection using a modified cardiac sympathetic index (CSI), based on the Lorenz plot. To consider everyday life activities, baseline exercise testing was included in the study to ensure that any positive results of testing could be compared to normal HRV episodes expected to occur in everyday life. The group tested four different methods of HRV-analysis with differing window lengths for each method to establish the best method. A patient specific threshold of 105% above maximum heart rate (including exercise) at any interictal period was used to establish seizure detection. The modified CSI with a window length of 100 s detected all seizures for 13 of the 17 patients, with either generalised or focal epilepsy, within a mean time of 16 s after onset ranging between 6 s before, and until 50 s after seizure onset time [35].

Fritz [36] showed that ictal-onset tachycardia occurred in more than 86% of 145 seizures of different epilepsy origin. Many cases also showed that heart rate increases occurred preceding EEG seizure onset. However, heart-rate-increase occurrence and amount differ significantly for different epilepsy types, length of effect and also general specificity of patients such as age and gender [37]. Further studies have shown that the FPR can drop significantly and more than 88% of seizures can be detected using an SVM classifier based on heart rate data alone [38].

### Accelerometery

An accelerometer (ACM) is a sensor that can track movement in the x, y and z plane. Accelerometry is useful in the detection of seizures, however, the seizures must take some physical form such as myoclonic, tonic-clonic or some other form of motor seizure. Other sensors such as gyros and magnetos, useful in measuring different forms of movement, can be used for detecting other seizure types and have their place in detecting movement associated with seizures [16].

Most detection systems use multiple accelerometers to ensure all areas that are potentially affected by seizures can be recorded. Recognising ACM patterns during seizures is relatively easy to distinguish by eye due to stereo typical patterns in different forms of motor seizure. However, the difficulty is finding the correct parameters to use in an automated algorithm [39]. A study conducted in 2005 [39] showed that 95% of seizures had stereotypical waveforms in the ACM-signals that could be distinguished by human observers.

Moreover, [39] also proposed a method utilising a model based matched wavelet (MOD) for detecting myoclonic seizures in ACM data with around 80% sensitivity and a positive prediction value (PPV) near 0.15. Furthermore, a method was proposed using a combination of 5 features extracted from accelerometry data that had an 83% sensitivity and a PPV of 0.35 for detecting tonic seizures in patients with severe epilepsy. However, it must be noted that 42% of the false alarms where seizures of another type [40].

An Australian study in using time domain features in controlled conditions reported an algorithm with 100% sensitivity with very few false positives (FP), however, the algorithm only considered seizure with >20s duration and was not validated in home conditions [41].

Other methods use as a wireless sensor network, and classification using the K-nearest neighbour (KNN) technique that showed promising results under normal daily conditions [42]. Other proposals include distinguishing nocturnal in-bed movements from epileptic seizures based on a hidden Markov model (HMM) [43]. Tests run on simulated epileptic seizures suggest that generalized models may be obtained using principal component analysis (PCA) and local PCA as feature extraction techniques using KNN classifiers [44], and an algorithm based on a Bayesian approach using HMM for statistical modelling tested on patients during the night, had good sensitivity but higher false alarm rates [45].

Most of these studies have been conducted on patients outside of normal everyday movement. Noise associated with normal movement and occurring from the surrounding environment, has yet to be dealt with, so the use of accelerometry to detect motor seizures as a viable all in one solution is not yet an option. However, its use as part of a larger hybrid solution may be viable.

### Local field potentials

There has been significant recent interest in the use of local field potentials (LFPs) as a biomarker for movement disorders such as Parkinson’s disease and essential tremor [46–48]. Discussions have not been so prevalent in regards to closed loop systems for epilepsy, however, there is evidence to suggest that LFPs could be a source of biomarkers for seizure prediction and detection [49].

Aibel [49] used microelectrodes inserted at temporal lobe regions, with wide bandwidth, high frequency recorders (0-6 kHz, 30ksamp/s, 10000 gain) while evaluating six mesial temporal lobe epilepsy patients during seven seizures. The recordings showed that clear epileptiform discharges could be seen in the LFP between approximately 3 s after and 20 s prior to seizure onset. Regarding hypersynchronous onset pattern seizures with ictal discharges < 2 Hz, prior to seizure onset fast ripples of increasing power occurred, they were also associated with an increase in the rate of ictal ripple and fast ripple, and individual spikes showed a ripple followed by a fast ripple. Although the sample size was small, the results were encouraging due to the similarity between seizures. Moreover, several animal models have recently been assessed to evaluate the potential of LFPs for seizure prediction and detection. Kainate-treated rats were used to evaluate the LFPs of the anterior nuclei of the thalamus (ANT) using the power efficient Generic Osorio-Frel algorithm to predict clinical seizures in TLE. From a total of 161 segments of ictal data and 103 segments of interictal data, the algorithm using LFPs from the ANT was able to predict the seizure onset with 100% sensitivity and 94% sensitivity with a FPR of 0.5 per hour in real time before the clinical onset of the seizures, and was preceding the prediction by the ECoG in 75% of cases [50]. Recently a study had some success in detecting seizures in zebra fish using only single channel LFP recordings in genetic and chemical induced seizures using both invasive and non-invasive LFP recording techniques [51].

A majority of LFP seizure detection models have yet to be clinically tested. Animal models have shown results that LFPs have potential to be a viable and perhaps even more accurate alternative to EEG based prediction/detection models. The works of [52] and later [53] suggest that applying brief pulse stimulation within 2 s of the occurrence of neuronal after-discharges (AD), used as a model for epileptiform activity, has 4.5 times more chance of stopping the AD. With this in mind, the latency values associated with LFPs could be useful to closed loop stimulation models.

### Video monitoring

Video monitoring is part of the gold standard for detecting seizures. Although used on its own, at this point, they have had limited use due to the requirement of the patient to be in the area of the camera to be effective, although this may be less of an issue during the night [16].

Video monitoring can use motion path methods based on tracking the space/time trail of moving objects. Velocity, area, angular speed, oscillation or rotation are some of the other values used to detect seizures by finding and interpreting motor patterns in video footage [14, 15].

Video methods can be marker aided or marker free. Marker aided methods require objects worn on joints or extremities which can be uncomfortable for the patient. Research in automated video analysis is limited, while progression has been made in recognizing various kinematic patterns in epileptic seizures, studies have been limited to recognising a limited set of seizures. It would be beneficial for progression to be made in a video system with a holistic view of epileptic seizures [54].

### Other methods

There are several other methods that have been studied for the prediction of epileptic seizures. A limited study consisted of inducing periodic electrical stimulation of cortex and assessing the intercranial EEG results using feature extraction and comparison in the interictal, preictal and postictal periods [55]. The study showed some interesting results that may result in further investigation, however, for the purpose of a closed loop neurostimulation device, the fact that it requires a constant although periodic stimulation may defeat the purpose of a closed loop system of this kind.

In 1997, [56] showed that there was a significant sustained increase in blood flow 10 min prior to a seizure in the epileptic temporal lobe, and at 2 min pre-ictus, an increase in both epileptic and non-epileptic temporal lobes. Another study discussed optical measurements of blood flow and oxygenation in animal and human neocortex. It concluded that the method could become important in predicting epileptic events [57]. The studies show that this method maybe of value in closed loop systems but maybe held back only by the availability and cost of wearable imaging devices.

Electromyography (EMG), a system that detects muscle signals, has been a subject of some studies that have resulted in wearable devices that have been on the market for some time. A study completed on one device showed a 91% sensitivity in detecting generalised tonic-clonic seizures in 11 patients within an average of 20 s of seizure onset [58]. Other proposals have shown mixed results when tested on other seizure types such as clonic seizures [59]. Although it has been found that EMG can produce reasonable results for certain seizure types and in certain muscle groups, currently it requires a specific type of seizure with specific placement to be of use as a one stop detection system, however, it has potential as part of a multimodal system.

Perhaps an important method of seizure prediction that has had less of a mention in previous reviews than it deserved, is self-prediction of oncoming seizures. A study conducted by [60] in 1997 focused on a questionnaire given to a relatively large group (562) of epilepsy patients with both generalised or focal seizures with a large demographic range. Patients were asked questions regarding warning and initial symptoms of epileptic seizures (WISE). Results showed that at least 47% of patients had prior warning of oncoming seizures, and the clear majority of these had at least 10 s warning until seizure onset. Further to this, a majority of those patients were able to commit spontaneous actions or follow instructions [60, 61]. found from a study of 49 patients that 67% experienced some form of aura before a seizure, and of these 85% could react during this time. However, [60] also found that there were also episodes where WISE occurred without a following seizure. This evidence shows there is potential for patient prediction of epileptic seizure, however, there is concerns that any seizure intervention method used maybe habit forming for the patient [60]. Therefore, there are ethical boundaries to cross, and the method may only be successful if used as part of a multimodal method.

Other methods include mattress detectors, seizure alert dogs, electrodermal activity and audio systems such as baby monitors. However, these methods are limited in use and would not be a focus for this application.

### Multimodal methods

Using multimodal methods have the potential to improve sensitivity and reduce the number of FPs in detection systems. These methods include combinations of those previously discussed, and include the same processes of feature extraction and thresholding or training processes that potentially provide a higher degree of resolution.

A MATLAB based software called EPILAB was designed and distributed as a free software for the use of researchers to perform studies in epilepsy prediction and detection. It allows the use of multiple algorithms to predict seizures from EEG data. It can be user adapted to allow the process of ECG/EEG data for prediction or detection of seizures [62, 63].

A study using 12 recorded (simultaneous) EEG and ECG (HRV) neonatal data, using a concatenate statistical based classifier model for seizure detection, was conducted with encouraging results. An independent and patient specific model was tested resulting in a 97.52% sensitivity, and 13.8% FPR for the patient specific model, while the results of 81.44 and 28.57%, respectively, were found for the independent model [64]. While this is encouraging, the dataset is limited to neonatal subjects, there is no mention of latency measurements and the greatest results were obtained in patient specific algorithms. In 2016, [65] developed an ECG/EEG method for seizure detection that was tested on 10 patients with diverse epilepsy symptoms from the EPILEPSIAE project [63]. It obtained 100% sensitivity and 99.91% specificity with an average latency of 2.6 s. Although there was a FP rate of 3/h, the method has the potential for online use, and testing on intercranial EEG data and a larger data base would be beneficial. Further testing could incorporate blood flow analysis or ACM data to lower False Positive rates and prediction analysis should be the focus of further studies.

Other methods proposed include combinations involving ACM, EMG or electrodermal methods to detect motor seizures. Although there is ongoing research into multimodal detection methods, there are a number of commercially available devices or devices that are still under testing that use a combination of these methods to detect seizures for patient warning systems [14]. However many of the commercial devices have no valid scientific data associated with them or are useful for only certain epileptic conditions [66].

Recently, a study conducted on a highly heterogeneous population of 69 epilepsy patients with nearly 6000 h of data, tested a model using a combination 22 ACM and 3 electrodermal features collected from wrist worn sensors. While patients engaged in some normal everyday activities, they did not engage in sports or physical labour, so while further testing in that environment is required, sensitivities of nearly 95% with a FPR of only 0.2 per day were achieved. However, the results show a median latency of 29 s [67], its use in online applications may be minimal and certainly for any closed loop neural stimulation application.

As a final example, [68] presents data obtained from simulated seizures using healthy patients to test a multimodal system made up of EMG, ACM and gyroscope data. The sensor system was integrated into a single suit. Although the suit was uncomfortable, even for healthy patients, due to the large number of electrodes, it produced some promising results for average sensitivity and FPRs. It was tested during everyday activities using wavelet transforms for feature extraction [68]. Although average latency was generally 1 s or below, more testing on real patients would be required to ensure the relevancy of these results.

## Control of refractory epilepsy

For those patients with refractory focal epilepsy that have either found resective surgery to be insufficient, impracticable or those that are fearful of the irreversible nature of these procedures, neurostimulation maybe a viable option to help control seizures. Moreover, as epilepsy is a chronic condition, it is encouraging that recent long term studies, even if all had some methodological problems, have shown most forms of invasive neurostimulation have had increasing efficacy over time with no adverse effects apart from device related complications [69].

An advantage of stimulation of focal epilepsies over AED systems that targets all areas of the brain stimulators are designed to target the area only where the seizure manifests [70]. However, the choice of stimulation methods will depend on whether the area of epileptic focus is well defined, or if it has several focal areas or extended regions, and where it is located. The patient’s tolerability is also an area a clinician must account for when choosing an appropriate stimulation method.

Table 1 compares an outline of three approved invasive stimulation devices for the treatment of epilepsy, a brief description of each stimulation method, and a review of its side effects.

**Table 1:** Approved invasive stimulation methods for epilepsy. Replicated from [70]

Data	Vagus nerve stimulation	Thalamic stimulation	Responsive focus stimulation
Approval	1997 (FDA)/(EU) (also heart rate triggered closed loop since 2015)	2011 (EU)	2013 (FDA)
Stimulation site	Left vagus nerve (neck)	Anterior nuclei of the thalamus (bilaterally)	Epileptic focus (cortex)
Stimulator placement	Subcutaneous, left pectoral/sub clavicular	Subcutaneous, abdominal	Within the skull
Stimulation mode	Open-loop/closed-loop based on detection of tachycardia	Open-loop	Closed-loop based on detection of ictal EEG patterns
Stimulus parameters	Intensity: 0.25–3 mA Frequency: 20–30 Hz Pulse width: 250–500 μs Duty cycle: 30 s on/ 5 min off (standard); 7 s on/30 s off (“rapid cycling”)	Intensity: 5 V Frequency: 145 Hz Pulse width: 95 μs Duty cycle: 1 min on/5 min off	Intensity: ∼1 mA Frequency: 200 Hz Pulse width: 160 μs Duty cycle: ∼5.9 min/day; (closed loop)
Side effects of implantation	1.6% infections 1% vocal cord paralysis	12,7% infections 10.9% local pain 18.2% paraesthesia at implantation site 4.5% in cranial bleeding	7.8% infections 4.7% intracranial bleeding
Side effects of stimulation	Hoarseness (intensity-dependent up to 66%) Cough (up to 45%)	14.8% depression 13.0% memory impairment	No statistics

### Vagal nerve stimulation

Vagal nerve stimulation has been approved by the US Food and Drug Administration (FDA) and in Europe since 1997 [70, 71]. The procedure involves implanting a pulse generator into the upper left side of the chest, and a lead is threaded to stimulate the left vague nerve at regular intervals by attaching the positive and negative electrodes to the nerve via a tethering anchor [72]. The procedure can be completed as either an inpatient or outpatient with either a general or local anaesthetic. The pulse generator is activated approximately 2 weeks after the procedure where the pulse is set at patient specific currents, frequencies and durations. In general terms these parameters are in the vicinity of: 1.0–2.0 mA, 500 μs pulse width, 20–30 Hz, 30 s ON, and 5 min OFF.

The maximum current is reached gradually using 0.25 mA increases. Moreover, patients are given a magnet that permits them to stop stimulation in case of complications or certain events, by positioning it over the pulse generator. Alternatively, they can deliver a single stimulation using pre-programmed parameters by passing the magnet over the battery [71, 72].

Although the exact mechanisms of VNS are not known, it is known that during VNS the unmyelinated type C fibers of the vagas nerve are stimulated. As described by [72], the device components are available as a kit and a sample of one such kit includes: (i) single pin Titanium-housed pulse generator with a Li/CFx battery (life of 6–8 yrs.), (ii) a 43 cm lead wire with two platinum/iridium helical electrodes and a helical tethering anchor, and.

(iii) a disposable subcutaneous tunnelizer. Most pulse generators today detect the heart rate for use in as a closed loop system [72].

In a retrospective study of 100 patients, [73] found that, on average, a half of patients found a 50% reduction, while a quarter found a 75% reduction in seizure. These findings were comparable with previously conducted studies. It must be noted that some patients found no change or worsening seizure rates following the procedure. Moreover, median seizure reduction trended upwardly over a 12 year period and although 50% of patients found side effects from the procedure, only 1 patient from 100 had side effects so intolerable, the device required removal [73]. Stimulation adverse effects include voice alteration, cough, dyspnoea, paraesthesia, headache and pain [74]. Patients receiving the treatment usually report improvement in mood, memory, cognition and general quality of life [72].

### Thalamic stimulation

There have been several studies conducted on intracranial stimulation with a focus on managing epileptic seizures. Some of the network areas focused on are: the cerebellum, subthalamic nucleus, centro median thalamic nuclei, caudate nucleus and hippocampus. Each of these areas have shown effectiveness in specific types of epilepsy [75]. While some of these studies have had results that are encouraging, enough to justify future larger studies, the bilateral deep brain stimulation of the anterior nuclei of the thalamus (ANT-DBS) is the only area, apart from the epileptic foci, that has gained widespread approval for stimulation [70, 76].

Like VNS, the exact mechanisms of action of stimulations are largely unknown. In a recent article, [77] suggested that high frequency ANT-DBS “may override the neural circuitry by blocking pathological activity and replacing efferent output”. There are several other suggestions for mechanisms of action and it is still a debated topic [77]. It has been shown previously, however, that high frequency stimulation can block epileptic activity in cerebral cortex while low frequency of below 1 Hz tends to synchronise activity [78].

Halpern [79] describes the implantation of the system by using an internal deep brain stimulation (DBS) pulse generator, generally placed in a sub clavicular pocket, bilaterally. The stimulation leads used during surgery are “DBS depth electrodes with 4 platinum–iridium stimulation contacts 1.5 mm wide, with 1.5 mm edge-to-edge separation because the ANT is relatively larger than other DBS targets” [79]. The lead is then secured to a burr-hole cap. A contralateral electrode is similarly inserted. Finally, an extension wire is tunnelled subcutaneously and attached to the leads and the implantable pulse generator [79]. Currently, a typical commercially available kit contains: (i) dual-channel implantable pulse generator kit and 7200 mAh battery (life of 5.8–10.5 years) including a magnetic switch, remote wireless programming, frequency programmable on each channel with up to 13 stimulation program storage, (ii) implantable lead kit for DBS, 1.27 mm diameter lead with 4 contacts, 1.5 mm spacing and length, 300 mm or 400 mm lead length, (iii) implantable extension kit for DBS, length 52 cm Includes anchorage and tunnelling tools, (iv) clinician programmer, (v) external pulse generator- for testing during surgical procedure, (vi) patient Programmer- for patient to change stimulation program as required and check battery life, and (vii) wireless remote programmer for clinician [80].

European approval for the procedure was granted following the randomized double-blind controlled trial of stimulation of the anterior nuclei of thalamus for epilepsy (SANTE) in 2010. The trial was extended to 5 years and involved 110 patients culminated with the release of results in 2015. The evidence presented was of class 1, however, there was a widespread variety of AEDs used during the trial. The trial showed that the procedure had a median seizure reduction of 41% at year 1, and 69% at year 5. Moreover, the responder rate, which is defined as a patient with a greater than 50% reduction in seizures, was 43% at year 1 and 68% at 5 years and the QOL in Epilepsy measure also showed significant improvement over the 1 and 5-year periods. There were minimal adverse effects over the trial period [81]. Recently, following the release of the 7 year data in 2017, the FDA approved the use of Medtronics ANT DBS System for use in the US [82]. Furthermore, there have been a number of other studies conducted that have given comparable results to these studies [83].

DBS of well-defined anatomic targets especially ANT-DBS has been shown to be effective in the short term and with greater efficacy over the longer term. To be effective they should be high frequency pulses of 5 V intensity although this can be a matter of patient specificity.

### Focal area stimulation

RNS is a system given FDA approval in 2013, after the results of a trial conducted in 2011. The RNS is designed for sufferers of focal epilepsy. It involves the electrical stimulation of up to two epileptic focus areas conducted by either depth electrodes or subdural cortical strip leads. The electrical stimulation is given in response to EEG patterns before ictal onset [83]. The intermittent stimulation is delivered to the epileptic focus area/s with the intention of limiting or inhibiting any abnormal electrical activity and preventing it from becoming clinical seizure [84]. Although this implies a closed loop system only, the mechanisms and results of the stimulation is discussed in this section. Unlike the ANT-DBS system the stimulator system is fully implanted in the skull. The neurostimulator is implanted in a burr hole in the patient’s skull and is connected to the electrodes via thin flexible wires. The electrodes are placed at the seizure onset zones, either bilaterally or at two onset zones depending on epilepsy type and can be placed in either deep structures (e.G. *hippocampus*) of the brain or on the surface. The four contacts on each of the electrodes can be predetermined as either anode or cathode, the stimulator case can serve as a cathode if required [84].

The electrical stimulation applied is biphasic, current controlled with high frequency. Typical stimulation parameters are between 1 and 3 mA, pulse width of 160 μs, pulse duration between 100 and 200 ms, and a frequency ranging between 100 and 200 Hz. It is noted that stimulation quantity is controlled by detection, however, compared with DBS, the total current density delivered remains low [70, 84]. The original works in this area, leading to this method, was conducted by [52]. The resulting work conducted on after discharges (AD) showed that the most effective stimulation was brief, between 0.5 and 1 s and needed to be applied within 4.5 s of the AD to abort it and similar bursts maybe effective on epileptogenic forms [53]. Controlled shorter-term studies, and uncontrolled longer-term studies, showed improved efficacy over time and improve QOL [85–88]. The results of the stimulation, however, almost certainly, have some connection to the system’s closed loop structure, so will not be discussed in this section.

### Non-invasive stimulation

Transcranial stimulation techniques such as transcranial magnetic stimulation (TMS) and transcranial direct current stimulation (tDCS) are techniques that can modulate cortex excitability and activity without the need for an invasive procedure. Moreover, other non-invasive techniques involving external stimulation of the vagas nerve or the trigeminal nerve are other areas that could deliver results in the control of epileptic seizures.

### Transcranial magnetic stimulation

TMS is a method of focal brain stimulation first described in 1985, where strong fluctuation magnetic fields created externally to the brain induce internal electric currents. TMS has several protocols that distinguish between pulse repeating length. In particular, low frequency (< 1 Hz) repetitive TMS (rTMS) has been tested as a means of reducing cortical excitability. Like most methods of stimulation for epilepsy, TMS has been found to be patient specific. Parameters such as frequency, intensity, type of stimulation coil, area of application, duration of stimulation, and the interval between trains have shown to differ significantly between patients [89, 90]. Risks associated with the use of this method have been found to be minimal, however, testing of the effectiveness of the treatment has produced mixed results, ranging from very, to not at all effective [89–91]. This may be due to the great variety in parameters used, and while the effectiveness is largely unknown, the non-invasive nature of the treatment warrants further investigation in controlled trials.

### Transcranial direct current stimulation

tDCS is a non-invasive method of stimulation that requires cortical direct current (DC), polarity dependent, stimulation. The stimulation has been shown to reduce or increase cortical excitability, dependent on polarity, for up to an hour after stimulation. The addition of certain medication can also help to prolong these effects [90]. To reduce seizures in epileptic patients, the general method involves placing sodium chloride solution-soaked sponge electrodes in specific positions on the head. The cathode is placed directly over the epileptogenic focal zone and the anode is placed over an area without epileptogenic activity contralateral to the stimulated side. Then a specific quantity of current at a certain frequency for a specified amount of time is applied. The procedure has generally been shown to be safe, well tolerated by the patient and relatively easy to apply [92]. Similar to TMS, clinical trials of the method have been limited, of mixed results, and have generally used different stimulation patterns [92]. A recent, small, sham controlled study conducted in 2017 showed a reduction in seizure activity [93], and shows that while trials of the method are in its infancy, there is evidence to suggest that larger controlled trials are required for what could be a beneficial, non-invasive therapy.

### Transcutaneous Vagus nerve stimulation

Recently, interest has progressed in a non-invasive method for vagal nerve stimulation. A small area of skin near the ear is innervated by the vagus nerve, and gentle stimulation has been shown to produce similar results in terms of fMRI brain activity changes as the surgically implanted device [94]. The method is generally patient specific, with changeable settings for frequency and current, with trials being conducted between 20 and 25 Hz, and approximately 1 mA. The length of time these currents are applied, and frequency of application are other parameters that can be disputed [95, 96]. This method of stimulation has been approved in Europe and the US for the treatment of a diverse range of conditions including one device for the control of epilepsy [74].

In the past, testing on epilepsy has had mixed results of efficacy and this again could be the result of the patient specificity of the method. However, results shown in studies such as the pilot study conducted on paediatric patients in China, and the double-blind randomised study conducted in Germany show encouraging results and good patient tolerability [95, 96]. These studies, coupled with the recent studies showing the increased efficacy with time of the surgical vagal nerve stimulation method, gives good cause for the need of future long-term controlled trials of tVNS for the control of epilepsy.

### External trigeminal stimulation

An alternative to tVNS is external trigeminal stimulation (eTNS). This form of stimulation is a relatively new therapeutic technique involving the stimulation of the trigeminal nerve. The trigeminal is the fifth cranial nerve [97], and like the previous methods, the exact mechanisms of action are relatively unknown.

Trials of eTENS for the control of epilepsy have generally involved using an external pulse generator connected to two electrodes placed above the eyebrows. In a 2013 trial [98] describes placement of electrodes that were “specifically designed to contact the right and left branches of the ophthalmic and supratrochlear nerves to provide bilateral stimulation”. Measured parameters used in trials were frequency, current, pulse width, with varying stimulation length times [98–100]. The frequency with greatest results in animal tests were > 100 Hz and so this was adopted in human trials, currents used varied but generally < 20 mA was used.

Studies that were conducted include a 6 month, 7 patient open proof of concept trial [99], an 18 week 50 subject double-blind randomized active-control trial [98] among others. Although results were mixed, they showed that the treatment was generally well tolerated, with little side effects relative to invasive methods, and they showed a general trend to improved efficacy over the time-period with an increase in mood and quality of life indicators [98–100]. The results show that larger multicentre controlled trials of eTENS would be advantageous for the pursuit of a non-invasive solution. Moreover, these trials have had similar parameter settings between patients, but due to the sometimes-large difference in beneficial parameter settings between patients, it may be advantageous to also take a patient specific approach during future testing.

## Closed loop neurostimulation

Systems involving DBS or other electrical based stimulation for the control of diseases such as Parkinson’s, essential tremor, depression among others have been found to be an acceptable form of treatment. Many of these systems use an open loop form of stimulation to continually induce stimulation to control what can be ongoing effects of the disease. However, epilepsy ictal periods occur after relatively long periods of interictal periods [101]. Although continual use of neural stimulation has shown to have minimal long-term side effects, other factors such as battery power and device wear could become problems with open loop systems due to continual use. In addition, other benefits could include early warning systems for prevention, by careers or patients, of secondary injury or sudden unexplained death in epilepsy patients. The ability to adjust levels of stimulation automatically if the current stimulation parameters are insufficient could also be beneficial [102]. Moreover, a permanent seizure detection or prediction sensor that records ongoing data could be vital for research, and further understanding of the disease and development of treatments. Currently, there are several commercially available devices available, and several suggested or trialled closed loop systems.

### Closed loop vagal nerve stimulation

In 2014, Cyberonics obtained CE mark and US FDA approved for use a vagal nerve stimulation therapy that utilises a closed loop invasive system based around the Aspire SR implantable generator. This technology was again upgraded in 2017 with the approval of the SenTiva model [103]. The therapy is a device that builds on the open loop VNS system discussed in the previous section. While the original system had some closed loop functionality in the form of a magnetic switch that activates stimulation at the behest of the patient or patients caregiver [102], the second generation pulse generator incorporates a sensor that implements a cardiac based seizure detection algorithm. Greater than 80% of epileptic seizures result in ictal tachycardia [36, 104] which is the basis of this system. The system can be useful for patients that have shown at least a 20% increase in heart rate and can be set to trigger using a predetermined threshold value to minimise the FPs stimulated by the system [104, 105].

A multicentre trial of the Aspire SR model involving 31 patients over 12 months showed the effectiveness of the cardiac based seizure detection algorithm and was highly patient and seizure specific. However, of those seizures that were detected, they were close to or even before ictal onset. Moreover, the trial showed that the effectiveness of the stimulation at the time of a detected seizure, could possibly reduce the severity of the seizure significantly [104].

A recently completed longer term study of 113 patients, with the Aspire SR system, had more encouraging results. The study involved patients under the care of one surgeon who had received new insertions of the model (51 patients) and those that had old VNS models that were updated at battery change (62 patients). The new (59% at a median of 13 months after insertion) and changed model patients both had better than expected responder rates over the period including a high rate of patients experiencing an additional seizure reduction in the changed model patients (71% had > 50% reduction in seizures following battery change) [106].

These results are mixed and somewhat complementary, however, this is most likely be due to technical difficulties in establishing the correct thresholds for the cardiac based detection systems and could depend highly on patient specific or seizure specific symptoms. What is encouraging, is the noted significant reduction in seizure severity when stimulation is applied before or during a seizure. This is a potential advantage that closed loop systems can bring to seizure control when opposed to open loop control. Open loop stimulations distribute stimulations repetitively for a pre-set duration and may not necessarily be applied during an ictal period to attempt to disrupt spontaneous epileptiform discharges.

### Responsive neural stimulation

There have been many small uncontrolled clinical and animal studies conducted on responsive stimulation for epilepsy over the last two decades. The studies have included targeting several different areas such as ANT, thalamic, cortical and epileptogenic zones with mixed results [107], however [108] is the only study in this area to produce class 1 results focusing on the epileptogenic zone. This study showed a mean seizure reduction of 37% during the 3-month follow up period, the results lead to the approvals for such a device in Europe and the US in 2013. This approval was gained for the commercially available RNS system designed and manufactured by NeuroPace. The stimulation system, described in a previous section, delivers pre-programmed electrical stimulation to up to 2 epileptogenic focus areas to attempt abortion of spontaneous epileptiform discharges. The key to success of this type of seizure prediction is in choosing the correct target area [108]. The depth electrodes or subdural cortical strip leads also act as ECoG recording instruments. The neurostimulator provides stimulation to the seizure focus once a seizure is detected. The system also provides wireless connection with the stimulator device for clinical programming and extraction of patient clinical data that can be uploaded to a database easily by the patient or carer [109].

The algorithms used for detection, utilises a choice of three tools, line length, bandpass and area to interpret the ECoG data [102]. The features are extracted, and the recent window of data is compared to the long-term trend, when the recent window trend is greater than the long-term trend by a predetermined percentage the stimulus is executed. The tool or tools used are patient specific in that they are utilised by how seizures typically manifest in terms of their ECoG recordings in a particular patient [107]. The tools are optimised so that real time detection requiring low computational power can be achieved within the constraint of the implantable stimulation device [102]. Once the pre-programmed stimulation pulse-train is delivered, the detection algorithm checks to see if the activity remains present and if so, up to an additional four therapies consisting of up to 2 pulse trains can be delivered, each of these can be of varying parameters [107]. A 7-year long term treatment study of 111 patients with mesial temporal lobe epilepsy that had undergone the RNS treatment showed, using a last observation carried forward method, a 70% reduction in seizures with 29% having seizure free periods greater that 6 months, the treatment was well tolerated by patients [110]. These results show good long-term efficacy for RNS treatment and are comparable to other long-term efficacy data for open loop.

### Other closed loop systems

Several closed loop systems have been tested both on animals and humans over the last two decades to find an appropriate system to initiate the various types of stimulation methods following real time detection of an identified seizure or epileptiform discharge.

These systems have both been invasive and non-invasive in nature, or a combination of both, and several recent attempts are interesting. In 2012, a team demonstrated a closed loop system using TES on rats that could be effective [101]. Studies conducted on the on-demand mode of VNS systems, where the VNS is initiated by patients or caregivers showed between 53 and 66% of patients reported being able to interrupt seizures themselves [111]. To improve on this, a clinical trial was performed that utilised the Digitrace 1800 Plus from SleepMed Inc. to collect EEG and ECG data. The system used the data from 5 patients with patient-specific algorithms to classify seizure or epileptiform discharges, activating an electromagnet positioned over the implanted generator to initiate VNS as required. The EEG or ECG data was used unconnected to distinguish patterns and initiate VNS. While the results showed good sensitivity, specificity and a short latency, it was suggested that fusing the datasets and even adding movement data such as accelerometry to the algorithm may produce a more sophisticated, accurate result [111].

## Discussion and future studies

Currently, there are several available systems that enable neurostimulation with a feature to control epilepsy for those patients whose options are limited. These devices are effective for a significant portion of seizures; however, the VNS closed loop versions yield similar results to open loop versions. Considering the studies described in this review, the large improvement in responder rates involving patients that received an upgrade to closed loop systems from their current system, there may be room to improve the VNS system by improving the detection algorithm.

Studies of the RNS system discussed in this review have not presented data indicating the effectiveness of the detection system used in the system. In the original works, the detection algorithms, described in [102], have the distinct advantage of being power conservative. They showed results in their development studies [112–114] that may now be inferior when comparing them to some of the newer studies presented in this review. With technological advances in processing ability, it could be suggested that there may now be room to improve on existing systems. According to ref. [108], the risks associated with the system’s use are comparative when taken in context with the risks associated with anti-epileptic drugs and are mainly associated with risks associated with any surgical implantation procedure. However, the benefits of having a full-time system recording brain activity similar to RNS are many. On an individual basis they potentially allow practitioners to make informed decisions of the patient’s progress and diagnose any changes in the epileptiform discharges. In a broader sense it can give researchers valuable data they have lacked in the past to possibly help better understand the condition and aid in design of more effective systems. Table 2 gives a description of the currently approved methods of stimulation available for epilepsy control, and gives an example of efficacy statistics that have been found in some studies. Furthermore, if applicable, it outlines the effectiveness of the detection algorithm employed in the system.

**Table 2:** Approved open and closed loop stimulation devices for the treatment of epilepsy and reported efficacy

Data	Vagus nerve stimulation	Vagus nerve stimulation	Thalamic stimulation	Responsive focus stimulation
Stimulation site	Left vagus nerve (neck)	Left vagus nerve (neck)	Anterior nuclei of the thalamus (bilaterally)	Epileptic focus (cortex)
Stimulator placement	Subcutaneous, left pectoral/sub clavicular	Subcutaneous, left pectoral/sub clavicular	Subcutaneous, abdominal	Within the skull
Stimulation mode	Open-loop 5 m off 30s on	Closed-loop based on detection of tachycardia	Open-loop 5 m on 1 m off	Closed-loop based on detection of ictal EEG patterns
Seizure reduction	^b^30.43% at 12 months 62.68% at 6 years [73]	^a^66% at 13 months [106]	^c^41% at year 1 69% at year 5 [81]	60% year 3 66% year 6 [87]
Responder rate	^b^49% at time of last follow up	^a^59% at 13 months [106]	^c^43% at year 1 68% at year 5 [81]	58% year 3 59% year 6 [87]
Effectiveness of detection/prediction algorithm	None used	No results have been given	None used	No results have been given

^a^Values are reported as mean values of the total new implant cohort

^b^Mean length of treatment time was 4.10 years with a maximum treatment time of 12 years

^c^Values reported are median values of 110 patients

The review on potential prediction and detection methods shows that progress has been made in EEG methods although there is a trade off with computational power required for more accurate predictions, and the ability to produce online results is questionable. Moreover, the non-invasive requirements could promote social stigma for the patient as well as comfort and.

practicality issues in using the equipment. Certain ECG and other reviewed methods lack accuracy, but are promising, such as HRV which produces a more sophisticated detection and prediction than a simple assessment based on heart rate thresholding.

Alternatively, the work involving LFPs is a relatively new and seems to be promising with animal models, and larger scale tests should be considered as an alternative to ECoG methods due to its less computational power requirement. However, the greatest leverage in these systems involves using hybrid methods of detection/prediction. Combining two or more carefully chosen methods from ECoG, ECG, LFPs, accelerometry and other methods has the potential to improve prediction/detection of seizures that contribute to a more potent closed loop system.

In terms of stimulation methods, they may potentially be under-performing. Open loop methods administer stimulation in repetitive doses not necessarily at the seizure manifestation. There is some evidence to suggest that issuing VNS at the commencement or during a seizure can stop or at least reduce the seizure. Focal stimulation is based on studies that indicate stimulation at the epileptogenic source can stop seizure manifestation. To fully evaluate its effectiveness, a system that can guide the stimulation to occur directly before and during a seizure 100% of the time is required. The same could be said for ANT stimulation and other regional stimulation systems under investigation.

## Conclusion

In conclusion, the ability to administer closed loop stimulation at seizure manifestation in a reliable and accurate way could potentially have a large effect on an epileptic patient, and their family’s quality of life. It has the potential to fill the gap for the 30% of 50 million epilepsy sufferers that are drug-resistant and have found no respite either in new AEDs or from surgical attempts to relieve seizures. Moreover, continuous recording of biomarkers and seizure activity can potentially help researchers with important clinical information that has not been available to them by traditional reporting and tracking methods. Current systems show they are quite effective for a large portion of patients by reducing seizure activity, however, the results show they are not yet at the level of sophistication and effectiveness to eradicate seizures in all patients.

## Data Availability

Not applicable.

## Abbreviations

ACM: Accelerometer
AD: After discharges
AED: Anti-epileptic drug
ANT: Anterior nuclei of the thalamus
ANT-DBS: Bilateral deep brain stimulation of the anterior nuclei of the thalamus
CSI: Cardiac sympathetic index
DBS: Deep brain stimulation
DC: Direct current
ECoG: Electrocorticography
EEG: Electroencephalogram
EMG: Electromyography
eTNS: External trigeminal stimulation
FDA: US Food and Drug Administration
FP: False positives
FPGA: Field-programmable gate array
FPR: False positive rate
HMM: Hidden Markov model
HRV: Heart rate variability
iEEG: Intercranial EEG
ILAP: International League Against Epilepsy
KNN: K-nearest neighbour
LFPs: Local field potentials
MOD: Model based matched wavelet
MSPC: Multivariate statistical process control
PCA: Principal component analysis
PPV: Positive prediction value
QOL: Quality of life
RLS: Recursive least squares
RNS: Responsive neuro stimulation
SANTE: Anterior nuclei of thalamus for epilepsy
SoC: System on chip
SRV: Support vector machines
tDCS: Transcranial direct current stimulation
TLE: Temporal lobe epilepsy
TMS: Transcranial magnetic stimulation
VNS: Vagal nerve stimulation
WISE: Warning and initial symptoms of epileptic seizures
